# Silent Invader: Cardiac Metastasis From Buccal Squamous Cell Carcinoma

**DOI:** 10.7759/cureus.64878

**Published:** 2024-07-18

**Authors:** Abier Atabani, Ahmed Basuoni, Abdallah Khreis, Syed Furqan Hashmi, Muhammad S Usmani, Layth Mula-Hussain, Zamzam Al-Hashami

**Affiliations:** 1 Medical Oncology, Sultan Qaboos Comprehensive Cancer Care and Research Centre, Muscat, OMN; 2 Cardiology, Sultan Qaboos Comprehensive Cancer Care and Research Centre, Muscat, OMN; 3 Nursing, Sultan Qaboos Comprehensive Cancer Care and Research Centre, Muscat, OMN; 4 Radiation Oncology, Sultan Qaboos Comprehensive Cancer Care and Research Centre, Muscat, OMN; 5 Nuclear Medicine, Sultan Qaboos Comprehensive Cancer Care and Research Centre, Muscat, OMN; 6 Radiation Oncology, Nineveh University, Mosul, IRQ; 7 Radiation Oncology, Dalhousie University, Halifax, CAN

**Keywords:** scc, buccal lesion, myocardial metastasis, myocardial infarction, cardiac mri, hnscc, cardiac metastasis

## Abstract

​Head and neck squamous cell carcinomas (HNSCC) are common malignancies that can metastasize to various distant sites. Cardiac metastasis (CM) from a primary HNSCC is an extremely rare finding that presents a significant challenge due to its association with a poor prognosis and limited treatment options. Due to their rare occurrence, there is no clear consensus on how to diagnose and manage such cases. In this article, we review a patient with complicated CM from buccal squamous cell carcinoma, which was incidentally detected by fluorodeoxyglucose positron emission tomography/computed tomography (FDG PET/CT).

## Introduction

The incidence of de novo metastatic head and neck squamous cell carcinoma (HNSCC) is around 10%, and up to 30% of patients with locoregionally advanced HNSCC will develop metastases during their lifetime [1]. In general, the prognosis of metastatic HNSCC is poor with median survival ranging between six and 15 months [1]. The usual sites of metastasis in such cases are the lungs, liver, and bones. Metastasis to the heart is exceedingly uncommon in such cancers, and often patients are asymptomatic, leading to a delay in diagnosis [2]. Most cases are detected in the postmortem setting [3]. Here, we elucidate the diagnosis and the outcome of a patient with buccal squamous cell carcinoma with metachronous cardiac metastasis (CM) leading to myocardial infarction (MI).

## Case presentation

A 54-year-old ex-smoker male, with a 30-pack-year smoking history, reported a lesion in the right inner cheek of two years duration. The lesion eventually progressed, and he developed ulceration and swellings in the neck. A biopsy of the lesion revealed moderately differentiated squamous cell carcinoma (SCC). Fluorodeoxyglucose positron emission tomography/computed tomography (FDG PET/CT) showed an irregular FDG-avid lesion in the right buccal mucosa, measuring 10x7x3.8 cm with extension into the right maxillary sinus, body of the hyoid bone, upper and lower gingivobuccal sulcus, and retromolar trigon. Infiltration of the buccinator and masseter muscles was noted. Multiple lymph node metastases were also identified in the right-level IB, IA, right retro-parotid, and deep cervical nodes. A small focal lytic lesion was seen in the body of the D10 vertebra. The clinical cancer stage was cT4a cN2b cM1 (IVA), as per the 8th version of the American Joint Committee on Cancer (AJCC) system [4].

The patient was initially managed elsewhere and was planned for surgical resection, followed by adjuvant concurrent chemoradiation to the primary site and stereotactic body radiotherapy (SBRT) to the D10 lesion. He underwent wide local excision of the right buccal lesion, right segmental mandibulectomy, infrastructure maxillectomy, right neck dissection, and a free anterolateral thigh flap. The following is the final pathological staging: pT4b pN3b cM1 (D10 oligometastatic disease) HPV-p16-negative SCC of the right buccal mucosa with mandibular erosion and involvement of masseter muscle, margins negative. Three out of thirty-six (3/36) cervical lymph nodes were involved with positive extra-nodal extension (ENE) and positive perineural invasion (PNI). He was then referred to our center for further management.

**Figure 1:**
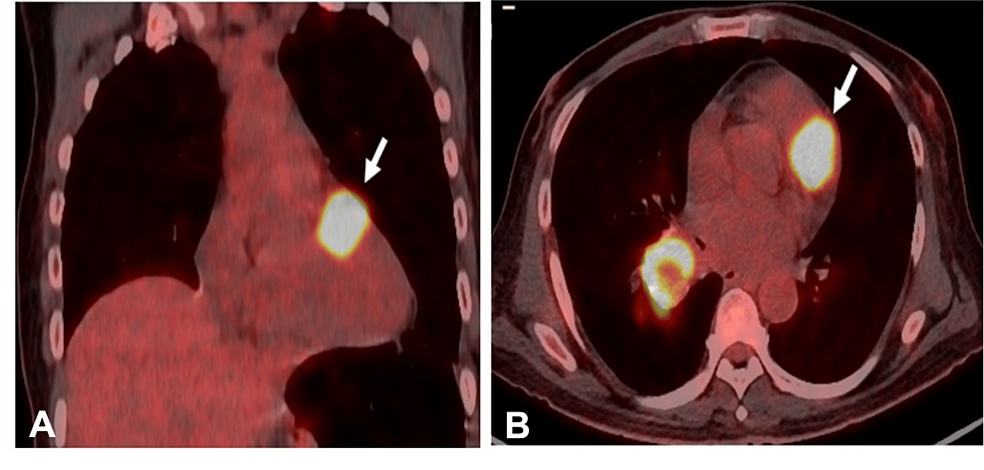
18-Fluorodeoxyglucose positron emission tomography/computed tomography (18-F-FDG PET/CT) images Sagittal (A) and axial (B) views of 18F-FDG PET/CT images showing intense FDG uptake of SUV max 15.1 (arrow) close to the pulmonary trunk.

Given the advanced disease stage on presentation, before initiation of adjuvant treatment, a restaging PET scan was performed six weeks after his surgery. This revealed multiple FDG-avid bilateral lung nodules, a right hilar lymph node, and an osteolytic lesion in the D10 vertebra. Hence, he was started on palliative intent chemotherapy with the TPEx protocol (docetaxel, cisplatin, and cetuximab) for four cycles and then maintenance cetuximab for six months till disease progression with multiple new bone and soft tissue metastases. A second-line immune checkpoint inhibitor, nivolumab, was given for another six months, after which he progressed with a new focal cardiac FDG uptake close to the pulmonary trunk, and a left adrenal lesion was identified on a follow-up PET/CT scan (Figure 1).

**Figure 2:**
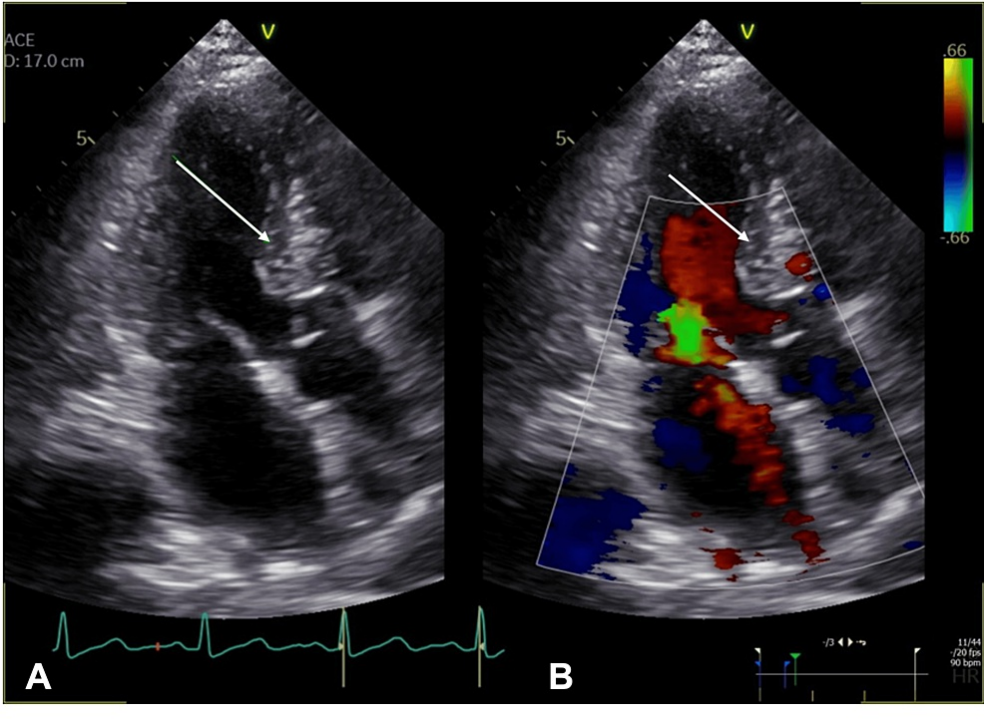
2D transthoracic echocardiogram (TTE) Apical three-chamber view without (A) and with (B) color Doppler showing a suspected mass (arrow) in the basal anteroseptal wall.

An electrocardiogram (ECG) revealed a normal sinus rhythm with no significant changes. A transthoracic echocardiogram (TTE) showed a mass in the basal antero-septal wall with normal left ventricular function and an ejection fraction (EF) of 68% (Figure 2).

**Figure 3:**
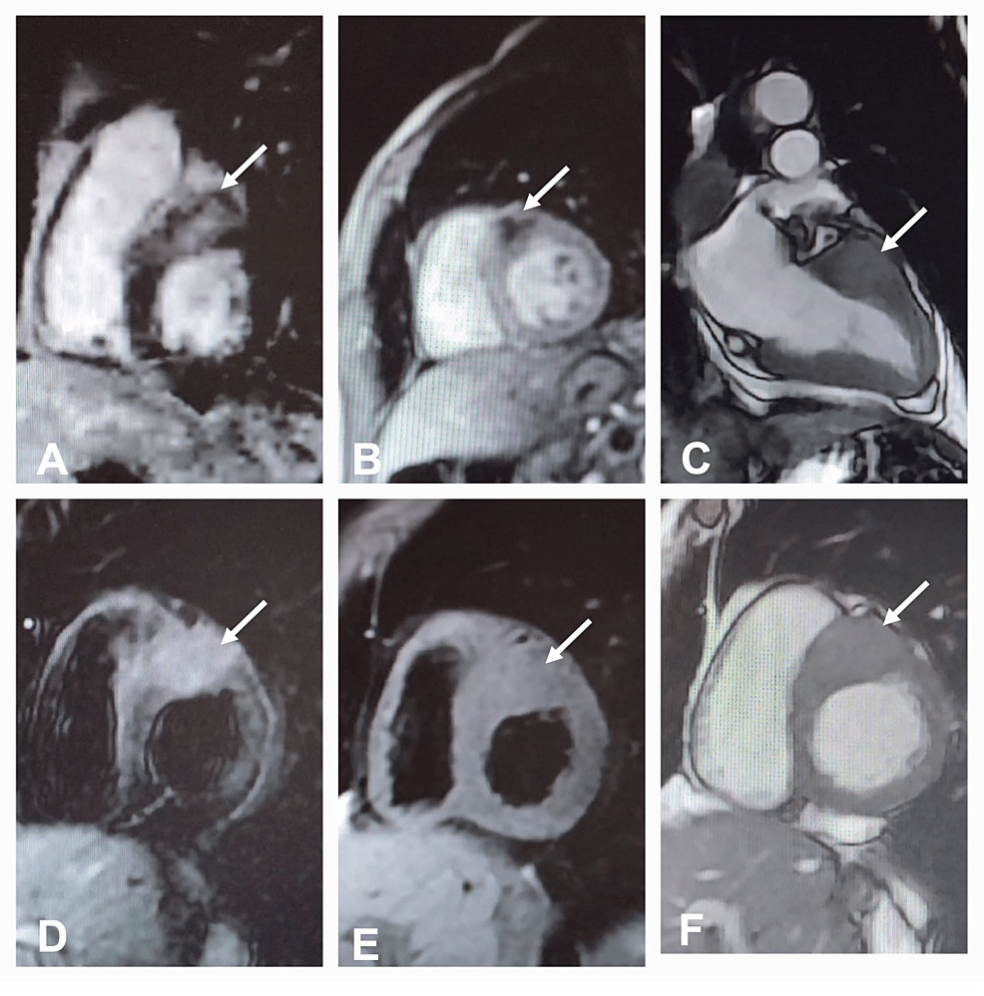
Cardiac magnetic resonance (CMR) sequences A: Late gadolinium enhancement, B: perfusion image, C: Cine 2-chamber view, D: T2 sequence, E: T1 sequence, F: Cine short-axis view. All images show a well-defined lesion in the basal anterior and anteroseptal wall (28x25x33 mm) with features in keeping with a metastatic deposit (isointense in T1, hyperintense in T2, a central non-enhanced component in perfusion image, and heterogenous enhancement in delayed gadolinium enhancement sequence).

Further evaluation with cardiac magnetic resonance (CMR) revealed a well-defined lesion in the basal anterior and antero-septal wall, measuring 28x25x33 mm with features that keep with a metastatic deposit (isointense on T1, hyperintense in T2, central non-enhanced component in perfusion image and heterogenous enhancement in delayed gadolinium enhancement sequence) (Figure 3).

Third-line chemotherapy with carboplatin and paclitaxel was given for four months. Repeated 18F-FDG PET/CT scan revealed disease progression in the liver, bones, and adrenal gland with a stable cardiac lesion (no change in size or FDG uptake). Due to the disease progression, the next line single agent gemcitabine was initiated, and he received one cycle.

**Figure 4:**
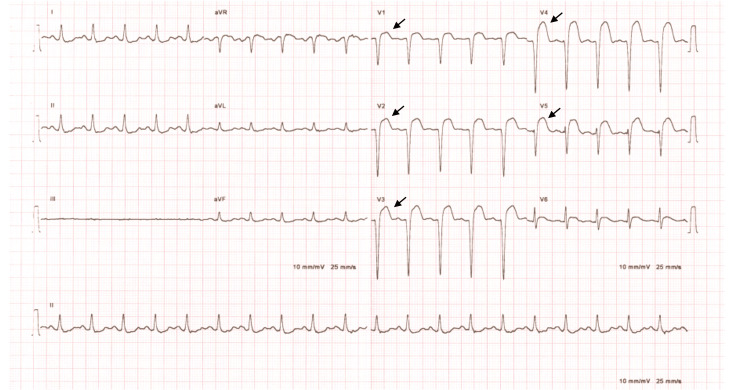
12-leads ECG showing ST elevation with pathological Qs in (V1, 2, 3, 4, 5) anterior and anteroseptal (completed MI)

He was evaluated in the daycare unit before the second cycle of chemotherapy, and he was found to have poor performance status with Eastern Cooperative Oncology Group (ECOG) 4. He had sinus tachycardia, with a heart rate of 122 beats per minute and a normal blood pressure of 118/68 mmHg. An electrocardiogram (ECG) showed a complete anterior and anteroseptal wall MI (Figure 4).

Serum troponin T was 409 ng/L (reference range: 0-14 ng/L). TTE revealed reduced left ventricular systolic function with an EF of 43% and basal to apical akinesia in the anterior and anterolateral walls. Due to his late presentation post-MI and poor performance status, he was not fit for further cardiac investigations and interventions. He was transferred under the care of the palliative team for best supportive care. He died peacefully one week after his infarction presentation (21 months from his initial diagnosis and 10 months from the diagnosis of cardiac metastasis).

## Discussion

Metastases to the heart are uncommon phenomena. Bussani et al. reported an incidence of 9.1% of cardiac metastases in postmortem patients with known cancer diagnosis, with only 5.3% due to HNSCC in contrast to 48.4%, 27.8%, and 21.0% secondary to mesothelioma, melanoma, and lung adenocarcinoma, respectively [3]. Distant oral squamous cell carcinoma metastasis occurs in 4.2-23.8% of patients during their disease course. The most common sites are the lung, bone, and liver, while heart involvement is uncommon [5,6]. CM from the oral cavity is higher than the larynx and nasopharynx [7,8]. Most HNSCC patients who developed CM during their disease course had an advanced stage of presentation.

The exact mechanisms underlying CM remain unclear; however, hematogenous spread, lymphatic spread, intracavitary diffusion, and direct invasion have been proposed as potential routes [3,5]. Across all tumor sites, the pericardium is the most common site in more than 60% of CM cases, followed by epicardium and myocardium [2,9]. However, in reported cases of HNSCC, metastasis to the ventricle/myocardium was the most common site compared to the pericardium [7], like our case. It is unclear why HNSCC has a propensity for ventricular/myocardial involvement compared to other primary malignancies.

Overall, most metastatic heart deposits are clinically silent and are often diagnosed postmortem, hindering early detection of these tumors [3,9]. In HNSCC cases, Dewan et al. reported that 61.1% (22 out of 36 patients) were symptomatic on presentation [7]. When they manifest clinically, the presentation can vary from common non-specific symptoms such as dyspnea, palpitation, and chest pain to more serious complications, including lethal arrhythmias, massive MI, and cardiac tamponade, depending on the location and the burden of metastatic deposit [9,10]. Myocardial metastasis can present with ST-T wave changes and high cardiac enzymes resembling acute coronary syndrome presentation, even in the absence of coronary artery involvement [5,11,12]. In our case, the patient was asymptomatic when CM was detected initially; however, later, he developed MI with ST-wave elevation in the ECG and high cardiac enzyme. Clinically, tachycardia was the only sign on presentation with no other cardiac symptoms. Such presentation is uncommon with few HNSCC cases reported in the literature [6-8].

Due to their silent nature and vague clinical presentations, cardiac metastases are often detected incidentally, necessitating further cardiac investigations and imaging. Echocardiography is the most widely used upfront modality to assess the heart. It is routinely obtained to evaluate the heart function, the valves, and any structural wall abnormalities or masses [2]. Its diagnostic accuracy can reach up to 80%, making it a good initial screening investigation [13]. However, cardiac CT and MRI are preferred for a detailed evaluation of the pericardium and extracardiac disease. Cardiac MRI provides specific cardiac details, including tissue characterization and the distinction of tumor from the myocardium or thrombus, in comparison with CT or ultrasound [10]. Furthermore, PET/CT is another imaging modality that may provide additional utility in detecting metastatic disease involving the heart [10,13]. Biopsy and histological examination remain the gold standard for diagnosis. However, given the significant risk of cardiac biopsy and the poor prognosis of such cases, it is rarely performed [7].

Treatment of metastatic cardiac diseases is challenging, and palliative systemic and radiation therapies have all been reported as treatment modalities based on the site of primary cancer, with surgery only indicated in exceptional cases [7,14]. In this case report, the patient had initially presented with de-novo metastatic disease and received palliative systemic therapy for 12 months after which he developed CM. Despite subsequent lines of palliative chemotherapy, he succumbed after 10 months of the detection of CM with MI, which most likely was due to the myocardial metastasis.

## Conclusions

Cardiac metastasis arising from primary oral cavity cancer represents a rare occurrence, necessitating the integration of multiple imaging modalities such as TTE, cardiac MR, and PET/CT scans for a precise diagnosis. Myocardial perfusion scans could serve as a valuable diagnostic tool to assess myocardial ischemia or areas at risk of developing infarction in patients suspected to have MI due to cardiac metastasis. The absence of clear guidelines for diagnosing and managing cardiac metastasis in head and neck cancer patients accentuates the complexity of the condition. Diagnosis proves challenging, as cardiac metastases often manifest silently, eluding clinical detection. Treatment strategies encompass chemotherapy, immunotherapy, and palliative radiation, with surgery reserved for exceptional cases. Overall, cardiac metastases in head and neck SCC entail a grim prognosis, underscoring the need for further research and refined therapeutic approaches in addressing this rare and challenging complication.
